# A rationale and framework for addressing physician cognitive impairment

**DOI:** 10.3389/fpubh.2023.1245770

**Published:** 2023-08-25

**Authors:** Victor A. Del Bene, David S. Geldmacher, George Howard, Catherine Brown, Elizabeth Turnipseed, T. Charles Fry, Keith A. Jones, Ronald M. Lazar

**Affiliations:** ^1^Department of Neurology, Division of Neuropsychology, Heersink School of Medicine, The University of Alabama at Birmingham, Birmingham, AL, United States; ^2^School of Public Health, The University of Alabama at Birmingham, Birmingham, AL, United States; ^3^Nursing Academic Affairs, Heersink School of Medicine, The University of Alabama at Birmingham, Birmingham, AL, United States; ^4^Department of Medicine, Heersink School of Medicine, The University of Alabama at Birmingham, Birmingham, AL, United States; ^5^University of Alabama Health Services Foundation, P.C., Birmingham, AL, United States; ^6^Department of Anesthesiology, Heersink School of Medicine, The University of Alabama at Birmingham, Birmingham, AL, United States; ^7^Department of Neurobiology, Heersink School of Medicine, The University of Alabama at Birmingham, Birmingham, AL, United States

**Keywords:** impaired physicians, aging physicians, cognition, neuropsychology, competence

## Abstract

Medical error is costly, in terms of the health and wellbeing of the patient, their family, and the financial burden placed on the medical system. Reducing medical error is paramount to minimizing harm and improving outcomes. One potential source of medical error is physician cognitive impairment. Determining how to effectively assess and mange physician cognitive impairment is an important, albeit difficult problem to address. There have been calls and attempts to implement age-based cognitive screening, but this approach is not optimal. Instead, we propose that neuropsychological assessment is the gold standard for fitness-for-duty evaluations and that there is a need for the development of physician-based, normative data to improve these evaluations. Here, we outline the framework of our research protocol in a large, academic medical center, in partnership with hospital leadership and legal counsel, which can be modeled by other medical centers. With high rates of physician burnout and an aging physician population, the United States is facing a looming public health crisis that requires proactive management.

## Introduction

As reported in 2022, an orthopedic surgeon and the hospital system where he operated, face approximately 350 lawsuits for hundreds of orthopedic injuries related to surgical error; all of which occurred while he allegedly performed surgery while known to be impaired (1). This striking case, although admittedly an extreme example, highlights the potential consequences of practicing medicine when cognitively impaired. Disruptive physician behaviors, such as cognitive impairment, increase the risk of medical error, patient harm, and litigation against the physician and the institution (2). Medical error is a broad term, encompassing diagnostic, surgical, prescription, and charting errors. Diagnostic error, as an example, is associated with hospital readmission (3) and is the leading cause of malpractice claims, ranking it first among both harm and severity to the patient (i.e., >50% resulting in death or permanent disability) and in financial cost (4). While it is impractical and impossible to eliminate medical error, attempts to mitigate error are warranted. Addressing cognitive impairment proactively with objective approaches, rather than subjective, is important to protecting patients, the physician, and the medical institution employing the physician. With an aging physician workforce, this will become a pressing issue for medical institutions in the United States and around the world.

A recent review of 67 studies identified 23 key factors that increase physician risk of malpractice, patient complaints, and impaired performance (5). When considering age alone, there was mixed evidence for increased risk of complaints at older ages. Impairment in younger physicians was associated with burnout, work-related fatigue, and alcohol abuse. Psychiatric illness and substance use were associated with poor outcomes in physicians 49–59 years of age. In physicians over the age of 59, the most common contributor to impairment was cognitive disorders. Physicians with cognitive disorders, regardless of etiology, are more likely to have unsolicited patient complaints, and medical error attributable to cognitive impairment have been found to increase throughout the course of successive shifts or with longer shift length (6). Fortunately, if burnout (7), sleep issues and fatigue (6, 7), depression (8), and substance abuse (9) are identified and treated early, potential harm to both patients and to physician livelihood could be avoided.

Late-career physicians have amassed significant knowledge and experience (10) but unfortunately, with increasing age comes increased risk for cognitive decline (11). As of 2020, approximately 30% of actively licensed, United States physicians are over 60 years of age (12). This raises several concerns. With late-career physicians retiring or working fewer hours, in the context of a US physician shortage (13), early– and middle-career physicians will need to absorb the increased workload (12), which in turn will likely increase burnout and mental health concerns that can adversely affect cognition and patient-centered outcomes (5, 7). While cognitive impairment can occur at any age (e.g., traumatic brain injury), the risk is elevated with increasing age. Across six hospitals, cognitive screening of actively working physicians over the age of 70 identified a 14.4% incidence of cognitive impairment (14). With advanced age, there comes increased risk for stroke, neurodegenerative illnesses (e.g., Alzheimer’s disease), and other systemic diseases, such as cancer (15–17), all which can result in cognitive decline and increases medical error risk. Moreover, as with their early-career colleagues, late-career physicians are also susceptible to burnout, fatigue, and psychiatric causes for cognitive impairment (7–9). An aging physician workforce, in conjunction with a physician shortage (13), is the perfect storm for over-burdened providers and risk for increased medical error (5) and thus, a potential, looming public health crisis (18).

In healthy, aging physicians, factors associated with health and well-being are more important predictors of cognitive decline than age alone (19), and cognitive impairment can be found across the career span (20). In addition to neurocognitive disorders, both untreated psychiatric illness and substance use can interfere with optimal cognitive functioning and increase the risk for work-related errors (9). Some causes of cognitive impairment are treatable (e.g., substance use, depression); therefore, it is essential to identify both the presence of impairment and the underlying cause, since successful treatment can potentially clear the physician to return to work. However, one of the biggest barriers to early identification of physician cognitive impairment is reluctance to self-disclose (21). In a survey of 1,000 surgeons, only 58% reported that they would stop performing surgery if they observed a decline, but in practice, 38% of the same sample reported they have noticed a subjective decline in their ability, but have continued to perform surgery (22). Of note, this study was a survey, making it difficult to ascertain the level of decline self-observed by the surgeon, but the trend is worrisome. Moreover, when a physician observes a colleague whom they suspect is impaired, there is reluctance or even unawareness of how to report their concerns to supervisors, administrative leadership, or medical licensing bodies (23, 24). There are likely many factors, including mental health stigma, fear of repercussions, denial, fear of being wrong, power dynamics, and personality traits, which mediate the reluctance to self-disclose or report such observations. These types of barriers preclude earlier proactive intervention.

Since their inception in the 1970s, physician health programs (PHPs) can be found in forty-seven states in the US. While there is controversy surrounding how these programs operate (25), PHPs were intended to identify and help physicians who are struggling with medical, mental health, and cognitive disorders (26). The most common cause of referral for a physician to a state PHP is a neurologic disorder (48% of referrals) (6). When a physician is referred for a neuropsychological assessment by a PHP, it is not uncommon for there to be divergence between their subjective report of cognitive symptoms and objective findings (27). The reasons for this discrepancy are likely variable across referred physicians, but may include anxiety or fear of potential repercussions, denial, and worry about the implications to finances or professional reputation. Furthermore, physicians are susceptible to the superiority bias effect, a disposition in which people with higher intelligence can overestimate their current abilities (28, 29). A literature review reported that physicians who performed worse on tasks of competency were those who were the most confident in their abilities (29). Therefore, since self-appraisal alone is often insufficient, objective neuropsychological assessment is needed if there are cognitive decline concerns.

Over the last decade, professional medical organizations in the United States (American College of Surgeons, American College of Obstetricians and Gynecologists, and American Medical Association’s Council of Medical Education) have advocated for cognitive screening (30). The Society of Surgical Chairs recommended mandatory cognitive and psychomotor testing of surgeons at least 65 years of age (31), and the California Public Protection and Physician Health Inc. published guidelines for the evaluation of late-career practitioners, highlighting cognitive screeners for these evaluations (32). In clinical practice, a common first line approach to evaluating cognitive decline is the use of screening instruments, such as the Montreal Cognitive Assessment (MoCA) or the Mini-Mental Status Examination (MMSE). In a review of cognitive screener use when evaluating physicians, Garrett and colleagues discussed the strengths and limitations of these screeners (28). They note that the sensitivity and specificity of these screeners are based on the performance of people with mild cognitive impairment (MCI) relative to healthy controls. They acknowledge that not every physician with cognitive decline, from their presumed or estimated baseline, will meet criteria for MCI, and individuals with small changes could be missed early in their cognitive decline trajectory. Further, the MoCA has norms that correct for level of education (33), but education correction appears to make the MoCA less sensitive (34). Relying on a cognitive screener alone can miss cases of true impairment, with 78% of acute stroke patients, for example, judged intact on the MoCA, but were found to be impaired in one or more cognitive domains on a neuropsychological assessment. In addition, 59% with a perfect MoCA score were found to have cognitive impairment on a more comprehensive neuropsychological assessment (35). Sensitivity concerns of these cognitive screeners have also been reported in other clinical populations, such as MCI related to Parkinson’s disease (36) and individuals with heart failure (37). Many cognitive screeners have ceiling effects (i.e., less measurement resolution at the higher end of a scale) that can reduce interpretability when evaluating those with higher levels of intelligence and cognitive abilities (38). In contrast to commonly used cognitive screeners, brief measures like the computer-administered MicroCog® and the Department of Defense’s Automated Neuropsychological Assessment Metrics (ANAM) have physician-based normative samples (28, 39), suggesting greater suitability for determining physician impairment. Despite medical societies calling for the use of cognitive screeners, we propose that brief, screening examinations lack both sufficient resolution and the normative standards to address the complexities associated with evaluating the cognitive function of practicing physicians.

However, what cognitive screeners lack, compared to more comprehensive neuropsychological assessments, is medical record review, clinical interview, a wider range of cognitive testing, and considerations of mood, personality, psychosocial, and other pertinent demographic factors (40, 41). Not to mention, screeners lack in-depth measurement of language competency and decision-making and are often limited in their assessment of memory. A strength of the neuropsychological assessment is the use of normative data that can adjust for pertinent factors (most commonly age and education in the general population). Using normative data, the neuropsychologist can calculate standardized scores and percentiles to determine if the performance is within normal limits relative to their baseline, as well as how far their score deviates from normative sample performances – the use of standard deviations and percentiles allows clinicians to determine if a score reflects normal within-person variability or a true change from baseline (41). Unlike a cognitive screener, the neuropsychological profile of scores, across a wide range of cognitive domains, can reveal the extent of impairment from baseline, along with patterns that can aid in the differential diagnosis process (41). Compared to cognitive screeners, the neuropsychological assessment is more sensitive and provides a more detailed analysis of a person’s cognitive strengths and deficits (40), all of which can help with determining if a physician is fit-for-duty.

It is well-accepted that the most comprehensive assessment of cognitive status in clinical populations is performed via a neuropsychological examination, in which multiple domains are evaluated including, but not limited to, language, memory, processing speed, executive function, decision-making, visual perception, and psychomotor skills. It is common that measures of mood are also administered; mental states, such as depression, are independent causes of cognitive dysfunction. When physicians are clinically referred or mandated by a regulatory body to complete a neuropsychological assessment, there are varying rates of impairment reported, ranging from 22 to 64% (30). One explanation lies in common practice within clinical neuropsychology to use age and/or education corrections (30). This approach is used because the usual goal is to determine whether individuals have cognitive impairment relative to others with similar demographics; thus, a 70-year-old is compared to another of similar age and not a 40-year– old, since it is known that normal aging affects some cognitive domains but not others (11). This approach, however, fails to address two important criteria in answering the question whether there is cognitive impairment in a medical professional. First, nearly all normative standards for neuropsychological assessment are derived from individuals in the general population. It is well-established that physicians, as a group, have significantly higher cognitive skills than the overall population (42). An average level of function in rapid decision-making in the population as-a-whole is unlikely sufficient for a physician working in an emergency room. Second, and more important, is that to determine whether a physician has impaired cognition, the standard should be a criterion level of cognitive competence necessary to provide appropriate clinical care, without reference to age or other demographic factors (30, 42, 43). Therefore, an initial step in achieving these objectives is to establish a normative cognitive database from a cohort of otherwise healthy, practicing physicians. Only then can a peer group establish the criterion levels of cognitive performance against which other physicians can be judged.

To date, only one study has published physician-derived, normative data on standard neuropsychological tests to evaluate physicians referred for cognitive evaluations (42). Although a step-forward to increasing the validity of such fitness-for-duty evaluations, the study’s reference group was comprised solely of a small number of urologists (*n* = 39). A larger sample of physicians is crucial to derive both a broader representation of medical specialties and to model more precisely the distribution of test scores with greater sensitivity and specificity. There are cognitive competencies of a psychiatrist, pathologist, emergency room physician, and neurosurgeon that very likely overlap, but there may be differences associated with specific disciplines.

## Study protocol objectives and design

### Primary objective

The primary goal of this study is to establish a normative cognitive database among physicians to further the science of physician fitness-for-duty. To achieve this goal, our team at the Heersink School of Medicine at the University of Alabama at Birmingham (UAB), UAB School of Public Health, and UAB administration united a broad range of stakeholders within the healthcare system to design a model to address these limitations. The principal investigator is Ronald M. Lazar, PhD, FAAN, FAHA, Evelyn F. McKnight Endowed Chair in Learning and Memory in Aging, Professor of Neurology and Neurobiology, Director of the UAB McKnight Brain Institute, and Director of the Division of Neuropsychology. In a first-of-its-kind partnership among physicians, hospital administrators, cognitive scientists, legal counselors, nurses, and biostatisticians, we have devised an initiative, funded by the University of Alabama Health Services Foundation General Endowment Fund. Once we have developed a normative cognitive database among physicians, representative of the broad range of specialties in an academic medical center, we will use this normative database to develop a set of cognitive standards, regardless of age, against which the performance of physicians can be ascertained empirically when the issue of decline arises. Discussed in greater detail later, we will use the Delphi process technique to determine the cognitive standards. The etiology of cognitive change is multifactorial and not pathologically-specific, so there are no prior assumptions, as to cause or treatability; the aim here is to determine solely whether a provider meets the standards of those practicing medicine.

### Participant recruitment, data collection, and analysis

In a protocol approved by the UAB Institutional Review Board, we are actively-recruiting healthy physicians, ages 35 to 65 years old, who are employed at UAB to participate in a non-clinical, neuropsychological assessment. We are recruiting 216 physicians whose practices fall within the following categories: (1) general medicine and mental health, (2) board specialists in medicine, (3) intensivists and interventionists, and (4) surgeons. These four groups were selected to broadly cover all medical specialties, while also accounting for differences in practice, such as performing a procedure.

To reduce bias in recruitment, our team received the names of every physician at the UAB Heersink School of Medicine. This list was then randomly ordered by the study biostatistician and then each physician was emailed in sequential order to reduce potential selection bias. Every physician at our institution has the same probability of being invited to participate. Since we could only recruit from our academic medical center, the findings are only generalizable to our institution. However, because of medical school standards in admission and training, the cognitive abilities of physicians across large clinical and research medical centers should be similar. No study has directly addressed this question though, and this may be a potential limitation. Prior to initiating enrollment, the study Principal Investigator (RML) presented the study goals at the Heersink School of Medicine department and division faculty meetings to share the study rationale, aims, and objectives. Our experience to date (*n* = 160) reveals 60% male, similar to the sex breakdown of UAB physicians. We are also observing the following age-breakdown: 35–39 (32%), 40–45 (20%), 46–50 (15%), 51–55 (12%), 56–59 (13%) and 61–65 (8%), following the same pattern of UAB physician demographics. We anticipate the demographics of the 56 remaining participants to be similar.

Enrollment began on January 1, 2022, and is planned to conclude October 31, 2023. Inclusion criteria include (1) physician currently employed by UAB or Alabama’s Children Hospital, (2) between 35 and 65 years of age, (3) fluent in English, and (4) healthy. Exclusion criteria include (1) a neurological history (i.e., traumatic brain injury, stroke, seizures, neurodegenerative illness), (2) a psychiatric history (bipolar disorder, schizophrenia, substance use disorders), and (3) ages outside of the 35-to-65-year-old age window. To increase real-world generalizability, we did not exclude physicians who are multilingual, or English was their non-primary language.

After physicians respond to the invitation to participate, they come to the Neuropsychology Clinic, written informed consent is obtained, and the neuropsychological tests are administered, taking 60–75 min to complete. These neuropsychological tests are commonly used in clinical practice and research (44), with standardized administration language and procedures. The tests and their respective cognitive domains are depicted in Table 1, and are administered in the following order: Rey-Osterrieth Complex Figure Test (RCFT) Copy, Animal Fluency, RCFT Immediate Recall, Hopkins Verbal Learning Test – Revised (HVLT-R) Immediate Recall Trials 1–3, Delis-Kaplan Executive Function Systems (DKEFS) Trail Making Test Trials 1–5, Wechsler Memory Scale – 3rd Edition Spatial Span, Grooved Pegboard Test, Judgment of Line Orientation (JLO), HVLT-R Delayed Recall, RCFT Delayed Recall, RCFT Recognition, Boston Naming Test (BNT), letter-guided verbal fluency (COWA), and the MoCA. For study purposes, we opted to balance breadth of cognitive domains and testing duration. To minimize physician hesitancy in participating in this process, out of concern that scores could be released to a regulatory or oversight entity, each participant is assigned an arbitrary study number. Tests are scored according to standardized criteria, and the raw scores for each participant are entered by a blinded research assistant into a REDCap® database containing the study number, age, sex, primary language, number of languages spoken, and group assignment, but no other identifying information. The test forms are then destroyed, so there is no mechanism to ascertain individual scores for any physician in the study.

**Table 1:** Cognitive domains and corresponding tests.

Domains	Tests
Processing speed	DKEFS TMT Trials 2 and 3 (Letter Sequencing, Number Sequencing)
Attention/Working memory	DKEFS TMT Trial 1 (Visual Search) Spatial Span
Language	Boston Naming Test Verbal Fluency (COWA /Animal Fluency)
Visuospatial	JLO RCFT Copy
Verbal memory	HVLT-R
Visual memory	RCFT Immediate & Delayed Recall
Executive function	DKEFS TMT Trial 4 (Letter-Number Switching)
Motor processing speed/Fine motor dexterity	DKEFS TMT Trial 5 (Motor Speed) Grooved Pegboard Test

DKEFS TMT, Delis-Kaplan Executive Function Systems Trail Making Test; JLO, Judgment of Line Orientation; RCFT, Rey-Osterrieth Complex Figure Test; HVLT-R, Hopkins Verbal Learning Test – Revised; COWA, Controlled Oral Word Association: individual to name as many words in 1 min that begin with the letters F, A, and S, respectively.

After completing the core cognitive test battery, we administer the MoCA to identify incidental cases of cognitive impairment, based on a pre-specified cutoff score. The MoCA is calculated at the time of the study visit. If a physician scores at or below the impairment criterion, they are notified immediately by a senior investigator and instructed to follow-up with their primary medical provider for further evaluation. For privacy purposes, the physician participant is requested to inform us that they have sought medical consultation, but are not required to disclose the outcome. As of June 14, 2023, none of the participating physicians have scored below our cutoff. Like the data from the larger test battery, the MoCA score is entered by subject number and the test form is destroyed.

At the end of data collection, our biostatistician will use the appropriate statistical methods to derive the mean and median values, along with the corresponding measures of variability (i.e., standard deviation, interquartile ranges). The statistics used will depend on whether the scores are normally distributed. Our primary analysis will be for the entire study cohort; secondary analysis will be for each of the four physician groups. There will be no correction for age, since the goal is to derive a set of standards appropriate for practitioners at any point in their careers.

### The Delphi technique

After our normative database is complete, we will bring together a panel of experts as part of a Delphi process. The Delphi Technique is a well-established approach in which experts convene to reach a consensus about an epistemic question (45). The goals of our Delphi process include: (1) identifying and understanding the problem of cognitive impairment in physicians, (2) identifying and understanding the current state of scientific research, (3) determining which cognitive abilities are most important for physicians broadly, and specialties more specifically, (4) understanding the strengths and limitations of our approach, (5) resolving controversial judgments, (6) identifying and formulating standards or guidelines for methodological and clinical interpretation, and (7) formulating recommendations for action. In our approach, we are planning on using two Delphi techniques. First, we will use a *Group Delphi technique* during which experts will be informed about our methods and findings, introduced to neuropsychological assessment, will be introduced to the goals of the Delphi process, and then as a group will discuss in real -time their judgments. This initial phase will be followed by an *Argumentative Delphi technique* during which justification for judgments will be defended by the experts (45). At both sessions, members of the research team will be available to answer questions pertaining to the study methods and findings, but the research team will not comment on the judgments of the experts. If a consensus is not reached by the end of the second meeting, a second Argumentative Delphi process with occur. The Delphi process will be managed by a trained expert from the UAB Office of Continuing Medical Education, which is independent from the research team.

For the Delphi process, it is important to invite experts with no known conflicts of interest. We will select experts by first reaching out to UAB Department Chairs to explain our strategy and goals. The Department Chairs will then solicit names of faculty members who are interested in potentially participating. Based on the names provided, we will invite physicians from all four groups with the widest range of ages, professional experiences, and diversity of sex and culture. Based on a recent review paper, there is wide variability in how many experts are included in Delphi approaches, ranging from three to 731 experts, with a median of 17 experts, with no clear consensus on an optimal number of experts (45). We aim to have 15–18 experts.

Minutes from each Delphi meeting will be kept, along with an audio recording to ensure accuracy and transparency. Outcomes of the process will be documented in a follow-up manuscript. We will report on the number and demographics of our experts, the number of Delphi rounds, and participation rate. For the establishment of thresholds for cognitive competency, our expert consensus target is 70%, with 60% often used as a threshold for agreement (45). We will also report on qualitative themes of both agreement and disagreement among the experts because this level of nuance will be critical to improve the clinical implementation of these normative data in fitness-for-duty evaluations, as well as furthering future research endeavors. After the normative database and threshold for cognitive competency are developed, the first application of these physician-derived norms will be in cases in which there is suspected cognitive impairment, based on performance during clinical practice.

## Discussion

In the United States, other professions, such commercial pilots, have age-related cutoffs imposed by the Federal Aviation Administration (FAA). The FAA, under the Fair Treatment for Experienced Pilots Act of 2007, raised the upper limit from 60 to 65 years of age (46). With regard to physicians, several other countries, including the United Kingdom, India, Japan, China, and Finland, have age-related retirement cutoffs for physicians (31, 47). While this approach reduces the risk associated with age-related illnesses (e.g., stroke, dementia, movement disorders) in late-career physicians, this approach faces challenges from the Age Discrimination in Employment Act (ADEA) (48). While age-based, mandatory cognitive screening (49) has been upheld in cases before US courts, Yale New Haven Hospital was sued for this practice (50) and was recently found to have insufficient evidence that a cognitive screening program improved patient safety (51). The ADEA only applies to employers and not state licensing boards (52), which can mandate neuropsychological assessment and make determinations whether a physician can safely practice medicine. We believe that the establishment of physician-based standards for cognitive competency provides a unique opportunity to empirically determine whether patient outcomes are affected by physician age and thus, the basis for mandatory assessment.

The process of a fitness-for-duty evaluation is highly stressful for the physician. To increase fairness and reduce psychological distress, which can also affect cognitive functioning, transparency and bias reduction are essential. In addition, there are multiple considerations, including due process, accommodations, and clinician support, that should be considered when implementing any formal program (52). From an ethical standpoint, our aim is to minimize patient harm, while simultaneously ensuring a fair and accurate appraisal of a physician’s cognitive function. Harm can come from the failure to allow an intact physician to return to work, as well as clearing an impaired physician who is at increased risk of medical error. Medical and neuropsychological societies are encouraged to develop assessment guidelines (42) and to fund further research into medical decision-making, causes of medical error, physician cognitive impairment with adjudicated outcomes, efficacy and optimization of physician health programs, and for rehabilitation treatments to provide a possible pathway to return to work.

In the context of a physician shortage, elevated provider burnout, and an aging, at-risk workforce, healthcare societies and medical institutions have a mandate to ensure that there is a sufficient number of cognitively competent physicians to meet patient and societal needs. The neuropsychological assessment is the most sensitive method to identify cognitive impairment, but existing normative standards are based on the general population and thus, lack the sensitivity necessary to make decisions about physician performance. Our model to obtain physician-based norms will increase the utility of physician cognitive evaluations, while ensuring the privacy of those peers whose data serve as the source of competency standards. This work will further the discourse on how to best manage and evaluate cognitively impaired physicians.

## Author contributions

RL is the principal investigator. VDB, RL, DG, GH, CB, ET, TF, and KJ all serve on the study workgroup. VDB wrote the initial draft. RL, DG, GH, CB, ET, TF, and KJ all provided substantial revisions and comments to the manuscript. All authors contributed to the article and approved the submitted version.

## Funding

This work was supported by the University of Alabama Health Services Foundation, P.C., General Endowment Fund, and the McKnight Brain Research Foundation (RML).

## Conflict of interest

The authors declare that the research was conducted in the absence of any commercial or financial relationships that could be construed as a potential conflict of interest.

## Publisher’s note

All claims expressed in this article are solely those of the authors and do not necessarily represent those of their affiliated organizations, or those of the publisher, the editors and the reviewers. Any product that may be evaluated in this article, or claim that may be made by its manufacturer, is not guaranteed or endorsed by the publisher.
